# Anesthetic Approach to a Case of Hepatoblastoma With Right Atrial Spread for Simultaneous Resection of Both

**DOI:** 10.7759/cureus.25796

**Published:** 2022-06-09

**Authors:** Protiti Chatterejee, Hariharan Subramanian, Sakthirajan Panneerselvam

**Affiliations:** 1 Anaesthesiology and Critical Care, Jawaharlal Institute of Postgraduate Medical Education and Research, Puducherry, IND

**Keywords:** transesophageal echo, right hepatectomy, pediatric cardiac anesthesia, anesthetic management, hepatoblastoma

## Abstract

Hepatoblastoma is the most common primary liver tumor in childhood. However, cases involving age >5 years are extremely rare. Invasion of the inferior vena cava and right atrium in hepatoblastoma places the patient in a high-risk group, and due to the rarity of such presentation, the preferred surgical approach is not clear. We present the perioperative anesthetic management of hepatoblastoma in an eight-year-old child with right atrial invasion at diagnosis, with no regression in size of the tumor after chemotherapy, treated subsequently with combined cardiac and liver surgery. Due to the possibility of impingement of the tumor thrombus onto the tricuspid valve or superior vena cava, or systemic embolization from the right atrium, access for the cardiopulmonary bypass was kept ready at the start of surgery. Intraoperative evidence of fragmentation of a small part of the right atrial tumor was noted in trans-esophageal echocardiography midway during left hepatectomy. This necessitated the emergency institution of cardiopulmonary bypass and en-bloc removal of the tumor thrombus with the remaining left hepatectomy specimen. The anesthetic management was further compounded by the risk of peri-operative pulmonary embolization, coagulopathy, blood loss and hemodynamic instability, ischemia-reperfusion injury, and post-operative hepatic, renal and pulmonary complications inherent in hepatectomies. The case presented a unique set of challenges to both surgeons and anesthetists. What was most evident from the successful management of such a case was the need for a team approach, with adequate communication between teams managing the patient.

## Introduction

Hepatoblastoma is the most common primary liver tumor and the third most common abdominal malignancy in childhood [1,2]. However, cases involving age >5 years are extremely rare, with an incidence of 0.1 cases per million children [3]. The Children’s Oncology Group (CLG) and Japanese Study group for Pediatric Liver Tumor (JPLT) advocate surgery for the treatment of hepatoblastoma. Invasion of the Inferior Vena Cava (IVC) and right atrium in hepatoblastoma places the patient in a high-risk group, and due to the rarity of such presentation, the preferred surgical approach is not clear. Previously reported cases have been managed with concomitant cardiac and liver surgery [4,5]. In other cases, cardiac metastases have completely resolved with chemotherapy, thereby requiring no surgery or less complex surgeries [6,7]. We present the perioperative anesthetic management of a case of hepatoblastoma with right atrial invasion at diagnosis, with no regression in size of the tumor with chemotherapy, treated subsequently with combined cardiac and liver surgery.

## Case presentation

**Figure 1:**
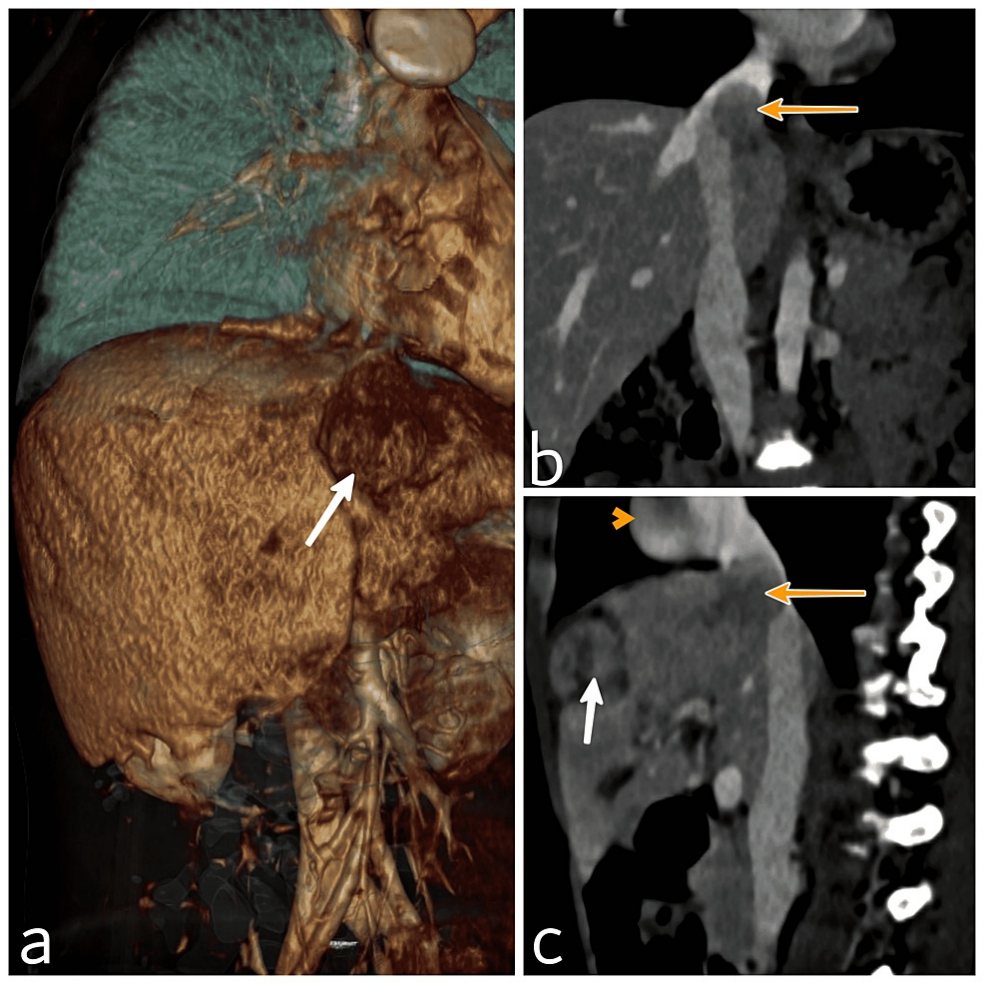
CECT images. (a) 3D volume reconstruction showing the tumor in the left lobe of liver (white arrow). (b) Coronal reconstructed section showing tumor thrombus in the IVC (yellow arrow), (c) Sagittal reconstructed section showing the tumor (white arrow), IVC thrombus (yellow arrow), and thrombus in the right atrium (yellow arrowhead). CECT - Contrast-enhanced computed tomography

An eight-year-old boy presented to the Department of Pediatric Surgery with complaints of gradually progressive epigastric swelling over a week, not associated with jaundice or fever. His blood investigations at presentation were normal apart from an alfa fetoprotein (AFP) of 81330 ng/ml. Contrast-enhanced computed tomography (CECT) of the thorax and abdomen revealed the presence of 7.6 x 6 cm heterogeneous soft tissue density mass supplied by the left hepatic artery, involving segments II, III, IVa of the liver with an exophytic component, presence of IVC and left hepatic vein thrombi, and possible thrombi in branches of the left portal vein (Figures 1a-1c).

The diagnosis of hepatoblastoma was confirmed with ultrasound (USG) guided core-needle biopsy of the liver lesion, following which the child received four cycles of PLADO (Cisplatin + Doxorubicin) chemotherapy. Post-chemotherapy AFP level was 2,595.1 ng/mL. Post chemotherapy CECT of the thorax and abdomen showed a 40% increase in the size of the lesion in the left lobe of the liver, causing compression and severe narrowing of the left branch of the portal vein. Enhancing tumor thrombus was noted in hepatic and supra-hepatic portions of IVC for a length of 3.7 cm, extending up to the cavo-atrial junction, with an increase in thrombus size noted as compared to his previous CT images. Thrombus was also noted in the distal part of the middle hepatic vein and its confluence with the IVC. Transthoracic echocardiography showed a 2.1 x 1.8 cm hyperechoic circular mass attached to the IVC-right atrium junction. The left ventricular ejection fraction was 60%, and the rest of the study was within normal limits.

He was subsequently posted for a left hepatectomy, followed by median sternotomy, cardiopulmonary bypass, and excision of the right atrial mass. His preoperative hemodynamics and blood investigations were within normal limits. There were no predictors of difficult airway on preoperative assessment. His weight on the day before surgery was 21 kg. On the day of surgery, two 24G intravenous cannulas were secured in the preoperative period. Two units of packed red cells and fresh frozen plasma were kept ready inside the operating room, and a team of cardiothoracic surgeons was made available inside the operating room for an emergency institution of cardiopulmonary bypass if the need arose during surgery. After an uneventful induction and endotracheal intubation, the left radial artery was cannulated with a 22G cannula. The right internal jugular vein was cannulated with a 5.5Fr triple lumen central venous catheter, under USG guidance, and with trans-esophageal echocardiography (TEE) monitoring, making sure that the tip of the catheter was well above the mass. Anesthesia was maintained with sevoflurane (minimum alveolar concentration up to 0.6) in an air: oxygen combination (fraction of inspired oxygen 40%). An initial dose of Tranexamic acid 250 mg slow intravenous infusion was administered to minimize bleeding. At inception, TEE was done and the extent of the right atrial lesion, the possibility of impingement onto the tricuspid valve or SVC, or systemic embolization of the tumor thrombus from the right atrium was assessed (Video 1).

**Video 1:** Intraoperative trans-esophageal echocardiographic findings of right atrial tumor mass

A consensus decision to perform sternotomy and keep appropriate access for emergency extracorporeal support was reached. Accordingly, a median sternotomy was done, the pericardium opened, and the right femoral vein at the inguinal region was kept ready for cannulation for cardiopulmonary bypass. Then laparotomy was done, and the left portal vein, left hepatic artery, and left hepatic duct were ligated sequentially, followed by separation of IVC from the left lobe of the liver. Intraoperative manipulation of the liver caused intermittent IVC compression and possible right atrial compression leading to sudden and drastic hemodynamic fluctuations, for which constant and vigilant monitoring was done. Repeat TEE showed that a small part of the atrial mass had partially fragmented and was attached to the parent mass by a stalk-like remnant. Hence, the hepatectomy was stalled and cardiopulmonary bypass was instituted after cannulating the aorta, superior vena cava, and right femoral vein. The IVC and right atrium were opened in a beating heart, and the tumor thrombus was removed en-bloc with the remaining left hepatectomy specimen. The total duration of surgery was 13.5 hours, and the total pump time was 67 minutes.

Before weaning off bypass, trans-esophageal echocardiography was done and the presence of residual intracardiac tumor thrombus or any valvular abnormality was ruled out. The child was shifted to the pediatric intensive care unit for post-operative management. Postoperative analgesia was achieved with an intravenous infusion of fentanyl. He was gradually weaned off inotropes over the next two days, his renal dysfunction, post-operative coagulopathy, and transaminitis were managed, and was extubated on postoperative day 2. Subsequently, he was shifted to the pediatric surgery ward on postoperative day 6.

## Discussion

**Figure 2:**
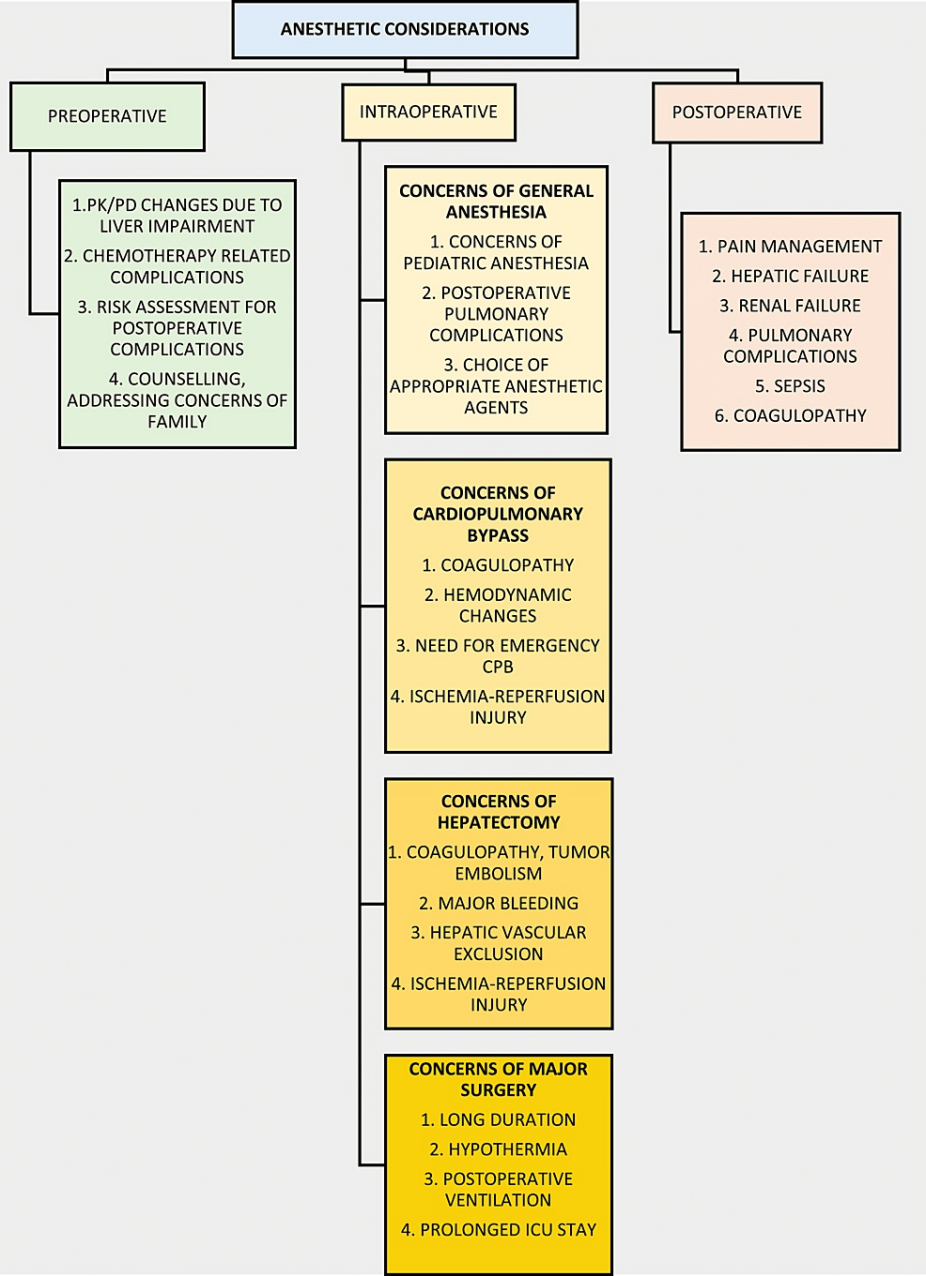
Chief anesthetic concerns for surgical management of pediatric hepatoblastoma with right atrial extension CPB: Cardiopulmonary bypass; ICU: Intensive care unit; PD: Pharmacodynamic; PK: Pharmacokinetic

Children posted for hepatectomy post-chemotherapy for treatment of hepatoblastoma present a unique set of challenges for the anesthetist. Added to that, our patient had a cardiac tumor whose excision was planned at the same sitting. This added to the complexity of our peri-operative management. The risk of peri-operative pulmonary embolization, coagulopathy, blood loss and hemodynamic instability, ischemia-reperfusion injury, and post-operative hepatic, renal and pulmonary complications inherent in hepatectomies were also compounded by the institution of cardiopulmonary bypass in our case. The existing literature database is lacking in descriptions of anesthetic management of such cases, hence creating a background for our case report. The chief issues we kept in mind during the management of the case are summarized in Figure 2.

During the preoperative period, our chief concerns were the alterations in drug metabolism and handling, complications related to chemotherapy including the possibility of difficult intravenous access, risk assessment for postoperative complications, and addressing concerns of the patient’s family. Placement of an epidural catheter for analgesia was avoided keeping the risk of coagulopathy in mind. Intraoperatively, provisions for emergency bypass in case of tumor embolism were made. Activated clotting time monitoring when on cardiopulmonary bypass was carried out keeping possibilities of coagulopathy in mind. The patient was monitored for hemodynamic changes due to hepatic vascular exclusion, mechanical compression of IVC or right atrium, ischemia-reperfusion injury, and massive blood loss [8,9]. The risk of sudden massive pulmonary embolism was a real threat with a possibility of sudden cardiac arrest. Postoperative issues of adequate analgesia, preventing hypothermia and managing renal and liver failure were also dealt with.

## Conclusions

Simultaneous excision of liver and cardiac tumors in the pediatric age group is a challenge to both surgeons and anesthetists. Apart from managing comorbidities, a thorough knowledge of the surgical plan and steps of surgery, and their anesthetic implications is required. Above all, of prime importance in the successful management of such cases is a team approach, with adequate communication between teams managing the patient.
